# New Aspects on the Structure of Neutrophil Extracellular Traps from Chronic Obstructive Pulmonary Disease and *In Vitro* Generation

**DOI:** 10.1371/journal.pone.0097784

**Published:** 2014-05-15

**Authors:** Astrid Obermayer, Walter Stoiber, Wolf-Dietrich Krautgartner, Michaela Klappacher, Barbara Kofler, Peter Steinbacher, Ljubomir Vitkov, Fikreta Grabcanovic-Musija, Michael Studnicka

**Affiliations:** 1 Department of Cell Biology, University of Salzburg, Salzburg, Austria; 2 Department of Pediatrics, Paracelsus Medical University, Salzburg, Austria; 3 Clinic of Operative Dentistry, Periodontology and Preventive Dentistry, Saarland University, Homburg, Germany; 4 University Clinic of Pneumology, Paracelsus Medical University, Salzburg, Austria; French National Centre for Scientific Research, France

## Abstract

Polymorphonuclear neutrophils have in recent years attracted new attention due to their ability to release neutrophil extracellular traps (NETs). These web-like extracellular structures deriving from nuclear chromatin have been depicted in ambiguous roles between antimicrobial defence and host tissue damage. NETs consist of DNA strands of varying thickness and are decorated with microbicidal and cytotoxic proteins. Their principal structure has in recent years been characterised at molecular and ultrastructural levels but many features that are of direct relevance to cytotoxicity are still incompletely understood. These include the extent of chromatin decondensation during NET formation and the relative amounts and spatial distribution of the microbicidal components within the NET. In the present work, we analyse the structure of NETs found in induced sputum of patients with acutely exacerbated chronic obstructive pulmonary disease (COPD) using confocal laser microscopy and electron microscopy. *In vitro* induced NETs from human neutrophils serve for purposes of comparison and extended analysis of NET structure. Results demonstrate that COPD sputa are characterised by the pronounced presence of NETs and NETotic neutrophils. We provide new evidence that chromatin decondensation during NETosis is most extensive and generates substantial amounts of double-helix DNA in ‘beads-on-a-string’ conformation. New information is also presented on the abundance and location of neutrophil elastase (NE) and citrullinated histone H3 (citH3). NE occurs in high densities in nearly all non-fibrous constituents of the NETs while citH3 is much less abundant. We conclude from the results that (i) NETosis is an integral part of COPD pathology; this is relevant to all future research on the etiology and therapy of the disease; and that (ii) release of ‘beads-on-a-string’ DNA studded with non-citrullinated histones is a common feature of in vivo NETosis; this is of relevance to both the antimicrobial and the cytotoxic effects of NETs.

## Introduction

Neutrophil extracellular traps (NETs) are web-like extracellular structures generated by activated polymorphonuclear neutrophils (PMNs) in a distinct process of cell death termed NETosis, involving the extrusion of nuclear DNA after chromatin decondensation. They consist of a scaffold of DNA strands of varying thickness laced with histones, neutrophil elastase (NE) and other antimicrobial, and potentially cytotoxic, molecules from the neutrophil cytoplasm [1]–[3]. Their fine structural morphology is characterised by two main components: smooth stretches with diameters of 15–17 nm and globular domains with diameters of 25–50 nm [4]–[6]. According to present evidence, NETosis can be initiated by a variety of molecular signals that bind to neutrophil surface receptors, among them bacterial breakdown products and endogenous pro-inflammatory inducers such as the chemokine IL-8 [2], [7]. A prominent feature of the reaction cascade leading to NETosis is the citrullination of core histones (mainly reported for histone H3) mediated by peptidyl arginine deiminase 4 (PAD4), likely a requisite for sufficient chromatin decondensation [2], [8], [9].

NETs have been shown to be effective in killing bacterial and fungal pathogens *in vitro* (eg. [10], [11] and *in vivo* on the lung mucosa, thereby interacting with surfactant protein D [12]. The antimicrobial activity appears to depend not only on the enzymes and histones, but also on the integrity of the DNA network, as it can be inhibited by DNase treatment [1], [13]. This is also a mechanism devised by bacteria as part of their strategies for escaping from NETs [14]. Knowledge on the effects and interactions of NETs *in vivo* is still fragmentary. Recent evidence from a bacterial infection model in mouse skin suggests that NET release *in vivo* occurs while the PMNs still move around by pseudopodial crawling, thereby casting large areas of NETs [15]. Evidence is accumulating that exaggerated NETosis and/or slowed NET clearance inflict tissue damage and organ function impairment in a broad variety of inflammatory diseases, including such affecting the human airways. It has been established that NETosis contributes to the pathology of allergic asthma [16] and especially of cystic fibrosis (CF) [5], [17]–[19], - and there is indication that NETs also play a role in chronic obstructive pulmonary disease (COPD).

COPD is an inflammatory disorder of the human airways which alternates between flare-ups (exacerbations) and periods of stable condition [20], [21]. It is characterised by recurrent bacterial infection [22], [23], neutrophil infiltration and emphysematous alveolar wall destruction [24]–[27]. The PMN-derived protease NE (a main constituent of NETs, see above) has been assigned a proinflammatory role in COPD, specifically by stimulating the secretion of IL-8 [28], the neutrophil chemoattractant which has been shown to drive NETosis in CF [17]. COPD is thus a prominent candidate for NET formation and NETosis-mediated tissue damage (cf. [1], [2], [6]), although there is as yet no clear morphological evidence.

The present study uses confocal laser scanning microscopy (CLSM) and electron microscopy (SEM, TEM) to establish the presence of NETs in sputum samples from patients with exacerbated COPD. Comparison with *in vitro* induced NETs from human neutrophils permits our results to expand the present knowledge on the micromorphology of these effector structures of innate host defence and inflammatory response.

## Materials and Methods

Investigations were carried out within the framework of a larger study on airway inflammation in patients with COPD at the University Clinic of Pneumology in Salzburg, Austria. Patients with COPD in acute exacerbation (thus their probability of having airway NETs is very high, n = 16) were recruited from the inpatient population of the clinic; COPD was diagnosed according to the GOLD guidelines [29]. Non-smoking control persons without airflow limitation (thus their probability of having airway NETs is very low, n = 15), among them one ex-smoker, were recruited through announcements in the hospital′s intranet and in local newspapers. Study groups are characterised in Table 1.

**Table 1:** Characterisation of study groups.

Study group	Age	Sex		smoking history (pack years)	FEV1 [l]	FEV1 [%]
	min-max (mean)	m	f	min-max (mean)	min-max (mean)	min-max (mean)
exacerbated COPD	46–87 (67.5)	11	5	5–80 (46.64)	0,50–1,60 (0.92)	14–57 (32.26)
non-smoking controls	41–77 (59.7)	7	8	0–15 (1.53)	1,81–5,39 (3.25)	93–129 (106.33)

### Ethics statement

All participants gave written informed consent before entering the study. The study was approved by the Ethics Committee of the Salzburg Province (full German name: Ethikkommission für das Bundesland Salzburg), Ref. No. 415-E/1171/12-2012.

### Sputum samples

Induced sputum was collected in a non-invasive manner with the help of an EasyNeb ultrasonic nebuliser following the protocols of the Eclipse study [27]. Harvested sputum samples were treated with 0.25 mg/ml Dithiothreitol (DTT) and - depending upon the method of further analysis - adhered to poly-D-lysine coated coverslips, poly-D-lysine coated Aclar strips, or Formvar-coated 200 mesh gold grids. A subset of the specimens on cover slips from each person was additionally treated with 2000 U/ml DNAse (30 min, at room temperature). All specimens were fixed in phosphate buffered 4% paraformaldehyde at 4°C.

### NETs generation from human neutrophils *in vitro*

For morphological comparisons, analyses were also performed on *in vitro* generated NETs from human neutrophils. Neutrophils were isolated from whole venous blood of consenting healthy controls (n = 4) using a standard gradient separation medium containing sodium metrizoate and Dextran 500 (procedure based on that of [30]). Harvested cells were resuspended to a concentration of 10^6^ cells/ml and adhered to coverslips, Aclar strips and gold grids as also used for sputum samples (see above). NETosis was induced by incubation with 1 µM formyl-methionyl-leucyl-phenylalanine (fMPL) for 2.5 to 3 hrs at 37°C. All specimens were fixed in 4% phosphate buffered paraformaldehyde at 4°C.

### Immunostaining for CLSM

Immunofluorescence staining for detection of citrullinated histone H3 (CitH3), neutrophil elastase (NE) and peptidylarginine deiminase 4 (PAD4) by confocal laser microscopy was performed as follows: Glass coverslips adhered with NETs containing COPD sputa (n≥3+ one DNAse-treated per tested person), or with NETs generated from human neutrophils *in vitro*, were washed in phosphate buffered saline (PBS, pH 7.4), blocked with 10% normal goat serum in PBS containing 10 mM glycine and 0.2% Tween 20, and incubated with rabbit anti-human CitH3 (citrullin 2+8+17) IgG (ab77164, Abcam, Cambridge, UK; 1∶50-1∶100), or rabbit anti-human NE IgG (Abcam ab21595; 1∶50) as primary antibodies. DyLight-conjugated goat anti-rabbit IgG (Abcam ab96883; 1∶100) was applied as secondary antibody; DNA was stained with propidium iodide (PI) (P4170, Sigma Aldrich, Germany). A subset of COPD sputum samples (5 persons, 2 coverslips each) was double-immunostained for peptidylarginine deiminase 4 (PAD4) and CitH3. Mouse monoclonal anti-PAD4 (Abcam ab128086; 1∶100) and anti-CitH3 (as above) served as primary antibodies; TRITC-labelled goat anti-mouse IgG (Abcam ab 6786; 1∶100) and DyLight-conjugated goat anti-rabbit IgG (Abcam ab96883; 1∶100) as secondary antibodies. DNA was counterstained with 4′,6-diamidino-2-phenylindole (DAPI) (Sigma-Aldrich, Schnelldorf, Germany). Normal polyclonal rabbit IgG (Sigma I5006) and PE-conjugated mouse IgG2A (BioLegend 400212) were used to replace primary antibodies in isotype controls. Specimens were analysed and photographed in a Zeiss LSM 510 meta UV CLSM (Carl Zeiss GmbH, Vienna, Austria).

**Figure 1:**
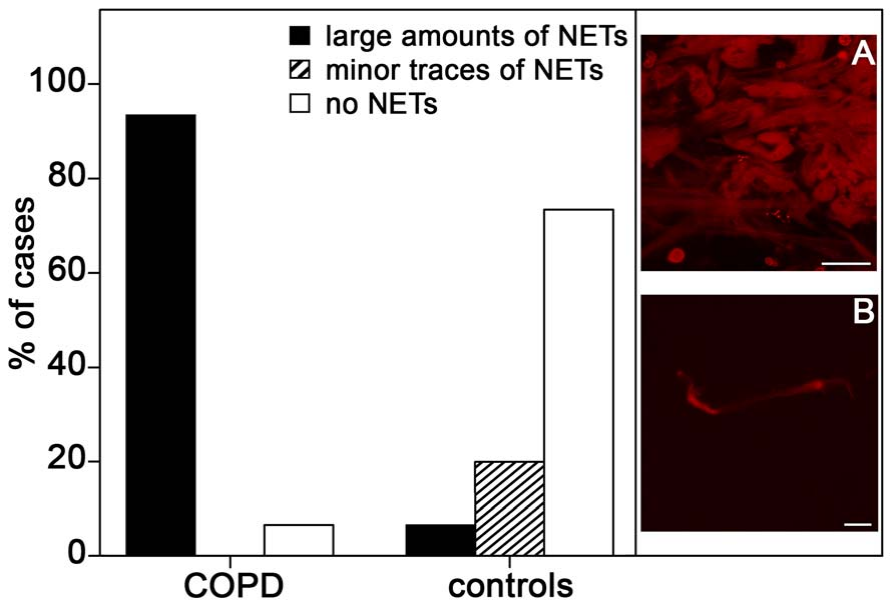
Semi-quantitative evaluation of NET abundance in induced sputa from hospitalized COPD patients with acute exacerbations and from controls. Images depict characteristic microscopic appearance conforming to the categories ‘large amounts’ (A) and ‘minor traces’ (B).

NE/PI-stained specimens (n = 3 per tested person) were used for semiquantitative evaluation. NET abundance was classified into two categories: (i) ‘large amounts’ defined as extended, confluent and/or overlapping formations with numerous neutrophils occupying at least about 1 mm^2^ coverslip surface area (in fact in nearly all cases more than ¼ of the coverslip surface) (Fig. 1, inset A), (ii) ‘minor traces’ defined as ≤10 small-sized (≤50 µm) non-overlapping items per 100 mm^2^, mainly associated with individual neutrophils (Fig. 1, inset B).

### Transmission electron microscopy (TEM)

TEM was employed in two sets of analyses. (i) Immunogold staining of sputa and *in vitro*-generated NETs on gold grids served to explore the ultrastructural localisation of CitH3 and NE protein within the NETs and their surrounding microenvironment. (ii) Samples collected on Aclar strips were employed to analyse the ultrastructural features of NETs and sputum components on ultrathin sections. Both sets of specimens were washed in PBS. On-grid specimens were subsequently blocked with 10% normal goat serum in PBS containing 10 mM glycine and 0.2% Tween 20, and incubated with the same primary antibodies as used for immunofluorescence analysis (see above). Goat anti-rabbit IgG conjugated with 5 nm colloidal gold (Abcam ab27235), diluted 1∶10 in Tris buffered saline (pH 7.8) containing 1% BSA, 0.2% gelatine, 0.2% Tween and 0.01% NaN_3_, served as secondary antibody. Finally, on-grid specimens were negative stained with a 1% aqueous solution of uranyl acetate (1 min, on ice). Aclar specimens were dehydrated in a graded series of ethanols and embedded in epoxy resin (Glycidether 100, Serva). After removing the Aclar strips from the polymerised resin blocks, semithin (1 µm) and ultrathin (80–100 nm) sections were cut from the fixed sputa on a Leica Ultracut 7 microtome. Semithin sections were stained with azure II-methylene-blue and viewed and photographed in a Reichert Polyvar microscope. Ultrathin sections were post-stained with aqueous solutions of 0.5% uranyl acetate and 3% lead citrate. All TEM specimens were analysed at 80 kV in a LEO EM 910 transmission electron microscope (Zeiss, Oberkochen, Germany) equipped with a Tröndle *Sharp:Eye* digital camera system.

### Scanning electron microscopy (SEM)

For SEM-analysis, specimens on cover slips were washed in PBS, dehydrated in an ascending series of ethanols, critical-point-dried and sputter-coated with gold. Specimens were examined in a ESEM XL30 scanning electron microscope (FEI Company, Eindhoven, Netherlands).

## Results

### CLSM analysis

**Figure 2:**
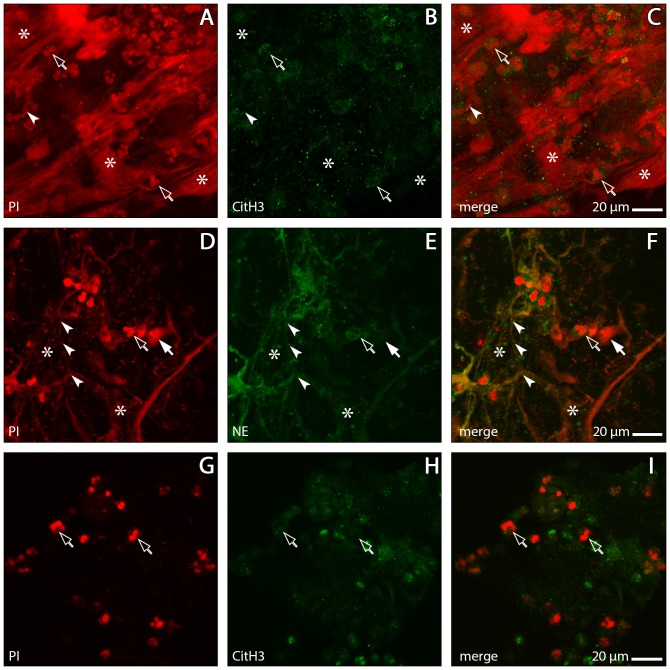
CLSM images of NETs in COPD sputum. Immunolabelling for neutrophil elastase (NE, green) or citrullinated Histone H3 (citH3, green), DNA stained with propidium iodide (PI, red). A–C: The sputum matrix contains PMNs (open arrows), citH3-positive granules and areas of condensed NETs (asterisks). D–F: Less condensed matrix traversed by thin NET strands associated with bacteria (arrowheads), non-activated PMNs (open arrow) and NETotic PMNs (solid arrow); asterisks indicate fully spread NETs. G–I: COPD sputum treated with DNAse. The DNA meshwork is dissolved while PMNs and citH3 positive granules persist.

**Figure 3:**
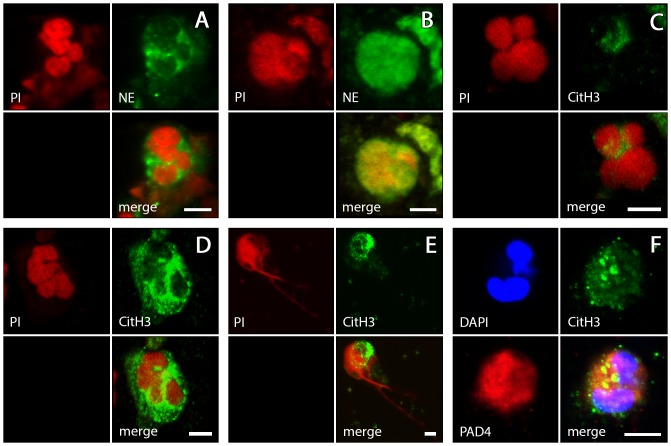
Individual PMNs in various stages of NETosis. COPD sputa immunolabelled for NE (green: A,B), citH3 (green: C–F), and PAD4 (red: F). DNA stained with either PI (red: A–D) or DAPI (blue: F). A: Non-activated stage with still lobulated nucleus, NE confined to cytoplasm. B: advanced stage with highly swollen nucleus, NE colocalised with chromatin (staining to the right extracellular). C–E: Stages during nuclear swelling. C: Nuclear lobulation still visible, part of chromatin stains for citH3. D: Staining for citH3 most intense in perinuclear cytoplasm. E: Chromatin extrusion. F: CitH3 and PAD4 colocalised in cytoplasm and nucleus. Scale bars are all at 5 µm and valid for each photograph within the individual combinations A–F.

Analysis with the confocal laser scanning microscope (CLSM) showed that sputa from exacerbated COPD patients contain extracellular DNA arranged in structures similar to those of in vitro generated NETs. In a very high proportion of these sputa (>90%; Fig. 1), DNA aggregates comprised of irregularly interwoven strings and ribbon-shaped strands are a dominant component of the sputum matrix (Figs. 2A–F). These DNA aggregates are frequently entangled with bacteria (as identified by their stained DNA, Figs. 2D–F), and always associated with PMNs, many of which are definitely recruited into the NETotic cascade according to previously established patterns [2], [13]. PMNs with slightly swollen nuclei (characteristic of the initial phase of the process) are routinely found, as are PMNs with disrupted nuclei and decondensated chromatin, and those that have entered chromatin extrusion (late phases) (Fig. 3A–F). The intracellular content of such PMNs is characterised by abundant granular particles that stain intensely for NE. Depending upon the stage reached in the NETosis process, NE is either separated from the chromatin and confined to the cytoplasm (undisturbed cells and early activated stages) or colocalised with the chromatin (advanced stages) (Figs. 3A,B). NE positive particles are also spread in large numbers throughout the sputum matrix where they are usually associated with the extracellular DNA (Figs. 2E,F). Somewhat surprisingly, staining for CitH3 in early activated stages was more common at cytoplasmic sites than at nuclear sites (Figs. 3C,D), but was usually co-localised with the chromatin of already extruded NETs (Figs. 2B,C). All of the subset of COPD sputa that were double-immunostained for PAD4 and citH3 contained numerous PAD4 positive PMNs. In most cases, PAD labelling was observed at nuclear sites and at cytoplasmic sites without any obvious co-localisation with DNA. Co-localisation of PAD4 and citH3 protein was frequent at both sites (Figs. 3F).

In DNase treated COPD sputa, all DNA aggregates were found to be dissolved, while PMNs and citH3 positive granules persisted (Figs. 2G–I). The sputa from healthy controls were, with the exception of a single person, devoid of NETs and activated PMNs (Fig. S1) or contained them only in minor traces (Fig. 1). Detailed clinical results will be presented in a separate publication. All negative controls displayed very low levels of unspecific antibody binding (Figs. S1,S2).

### Ultrastructural analysis

**Figure 4:**
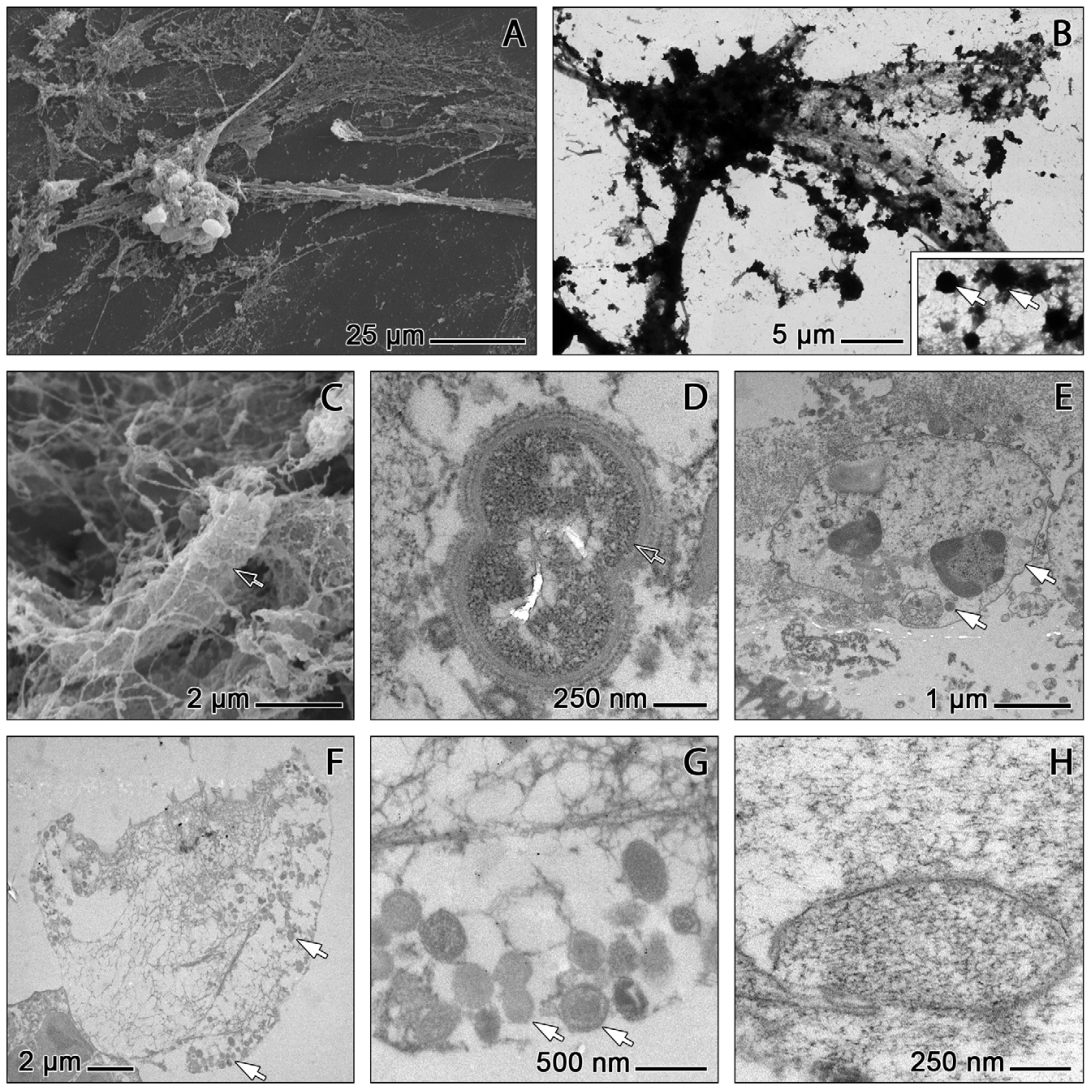
Electron microscopic characterisation of NET components. SEM (A,C) and TEM (B,D–H) micrographs from COPD sputum (A–E,H) and *in vitro* induced NETs (F,G). A: Fibrous strands diverge from a PMN cell body with a sculptured surface and attached granulae and merge into loosely structured texture. B: Motif corresponding to that of A. Thinly spread areas (inset) exhibit a fibrous texture with attached globules (arrows). C: Bacterium (open arrow) entrapped by NET fibres with globular protrusions. D,E: Sections of COPD sputum. D: Bacterium (open arrow) surrounded by a fibrous network that embeds spherical granules with amorphous content (arrow). E: Undisturbed PMN with normal nuclear chromatin and amorphous vesicular inclusions (arrows). F–H: Sections of *in vitro* induced NETs. F: NETs attached to the remnants of their cell of origin. NET fibres exhibit less accretions than those from COPD sputum NETs (cf. E); arrows indicate neutrophilic granula. G: Fibres and neutrophilic granula of the NETs shown in F. H: Membrane coated in COPD sputum vesicle containing NET-like structures.

On low magnification SEM and TEM images, COPD sputa appear as a meshwork of interwoven strands of varying dimensions and orientation. The meshwork comprises aggregations of condensed matter and more loosely structured areas which may be traversed by thick straight strands (Figs. 4A,B). The condensed areas usually accumulate pieces of cell debris, granular structures, and cells of rounded shape with locally protruded (‘blebbed’) or ruptured outer membranes. Such cells frequently constitute major ‘grid points’ within the meshwork from which large straight strands radiate. Their morphology is consistent with the well established appearances of PMNs under the SEM [1], [31]. The fibrous network around these cells is usually very dense, and many of them were, most probably, fixed in the process of NETosis (Figs. 4A,B). The loosely structured areas of the mesh consist mainly of small fibres at random orientation. These fibres are entangled with bacteria (Figs. 4C,D), various pieces of cellular debris, some (mainly intact) PMNs, and with granula in the size range of 200–900 nm (Fig. 4B). Under the TEM, these granules appear homogeneously stained at medium densities. They are similar to the vesicular inclusions contained in intact PMNs (Figs. 4E) and in PMNs at advanced stages of NETosis. In the latter, packs of such vesicles can be found within the remains of the cell membrane, while others are associated with the extruded fibrous mesh, thus supporting their identification as neutrophilic granula (Figs. 4F,G). This applies also to membrane coated vesicles with a lighter stained inhomogeneous content (Fig. 4H) which resemble a type of vesicles found in human neutrophils during bacterial-induced NETosis [32].

**Figure 5:**
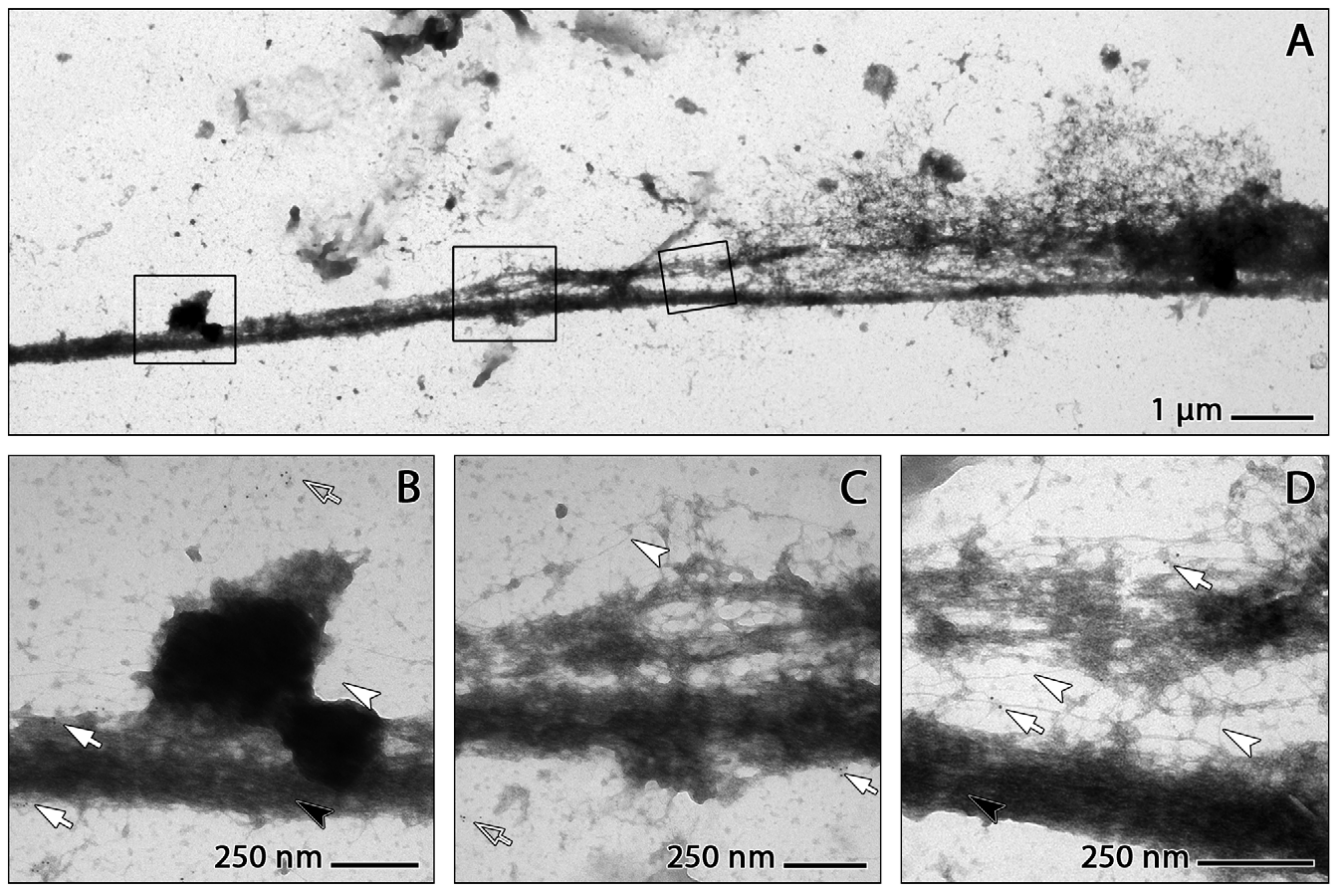
Ultrastructure of *in vitro* generated NETs. TEM images of *in vitro* generated NETs immunogold stained for citH3. A: Thick NET trajectory with attached electron-dense substance and small branches giving rise to loosely structured fibrous meshwork. Frames depict details shown in B–D. B: Patch of dense substance adhering to a thick NET strand with parallel fibrous subarchitecture (black arrowhead). C,D: Examples of NET strand disintegration into a network of randomly oriented small fibres with diameters down to about 2 nm (arrowheads). Some accretions at fibre intersections and along the strands are labelled for citH3 (arrows).

**Figure 6:**
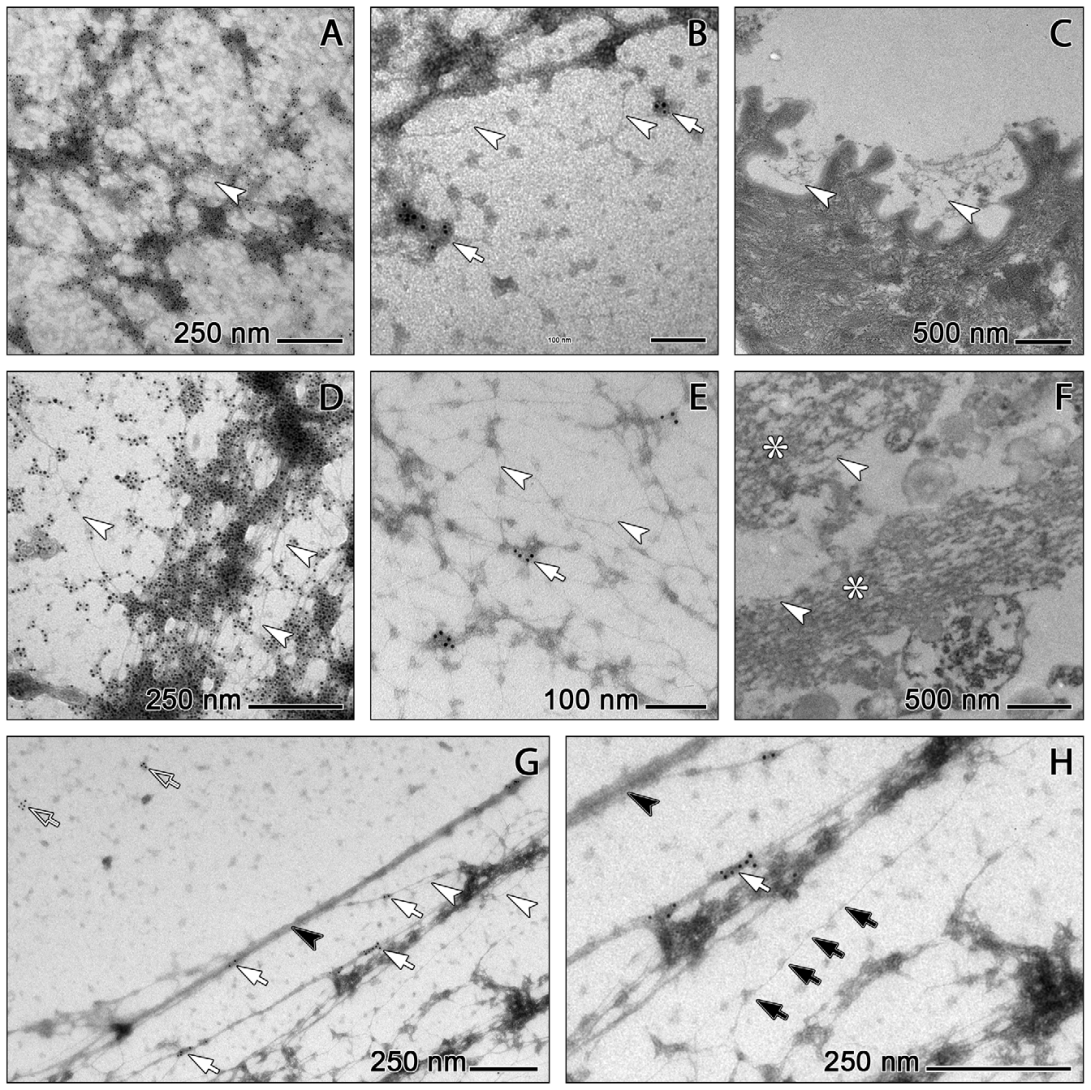
Detailed TEM analysis of NETs from COPD sputum (A–C,F) and *in vitro* generated NETs (D,E,G,H). A,D: Immunogold staining for NE. Amorphous attachments and ‘grid-point’ aggregates of both COPD NETs (A) and *in vitro* NETs (D) are all strongly labelled. B,E: Immunogold staining for citH3. Only some of the aggregates adhered to COPD NETs (B) and *in vitro* NETs (E) are stained (white arrow). Arrowheads indicate fibres consisting of single DNA double-helices. C,F: Sectioned COPD sputum. C: NET attachment to epithelial cell surface. F: Individual NET fibres (arrowheads) and bundles of such fibres in parallel alignment (asterisk) interspersed with granules and pieces of cell debris. The bundles are abundantly clotted with amorphous substance. G,H: Structure of fully spread NETs. Bundles of parallel fibres (black arrowheads) provide a scaffold for an irregular network of much finer fibres with diameters down to about 2 nm (white arrowheads) some of which are equidistantly decorated with protrusions of about 10 nm, likely corresponding to nucleosome cores (black arrows in H). Immunolabelling for citH3 recognises sites along the fibrous bundles (white arrows) and on detached substance (open arrowheads in G), but in no case the presumed nucleosomes.

The TEM analysis also provides detailed information about the structure and organisation of the fibrous components of the mesh. The thick strands of the mesh are in fact clusters of thinner fibres at parallel orientation. These are, however, partly clotted with amorphous matter (Figs. 5A–D). At the sites where the thick strands disintegrate (like the yarns of a rope) to form a more finely spread mesh, they are frequently associated with small globular protrusions which may also be clustered to form larger aggregates. These protrusions correspond in form and size (about 25 nm) to the 'globular domains' of NETs described by Brinkmann and coworkers. [1] (Fig. 5C). Under the TEM, these protrusions appear less rounded than under the SEM (Figs. 5D, 6A–E).

An important aspect is that the finest fibres of the mesh have diameters down to 2 nm. This corresponds to the size of single DNA double helices [33]. These 2 nm fibres are partly associated with a further class of globular structures which are smaller than the ‘globular domains’ of Brinkmann et al. [1]. They have diameters of about 10 nm and decorate the fibres in a beaded manner (Figs. 6G,H). Such beaded single fibre stretches bear a high similarity to the nucelosome-studded ‘beads-on-a-string’ fibres known from spread chromosomal DNA (eg. [33]–[35]). They have been most clearly depicted in NETs formed *in vitro* (Figs. 6D,E,G,H), but are definitely also present in sputum-derived NETs, although more frequently masked by accretions of sputum derived matter (Figs. 6A,B,F).

Immunogold labelling allows a precise localisation of NE and citH3 within the mesh and its cellular constituents. Most of the aggregates at the ‘grid points’ of the mesh (i.e. the above mentioned ‘globular domains’) stain for NE (Figs. 6A,D) while only a minority of these structures are positive for citH3 (Figs. 6B,E,G,H). NE and citH3 positive particles are also regularly found detached from the fibrous mesh, where NE is again clearly more abundant than citH3 (Figs. 6D,G). Interestingly, the globular structures identified as nucleosomes on ‘beads-on-a-string’ DNA (see above) never stain for citH3 (Fig. 6H), indicating that their histone complexes are not citrullinated.

## Discussion

The results of the present study deliver direct new morphological evidence that NETs are a major component in the sputa of patients with exacerbated COPD. Equally importantly, the present results also provide new knowledge of the structure of NETs and their possible biological functions.

The combined evidence from CLSM, SEM and TEM strongly supports the conclusion that relevant amounts of the PMNs known to invade the airways of COPD patients undergo NETosis, thereby generating functional NETs, specifically during the exacerbation episodes. Accordingly, the sputa of such patients were commonly characterised by the pronounced presence of both NETs (Figs. 2A–F, 4A–E,H, 6A–C,F) and NETotic PMNs (Fig. 3), in strong contrast to the absence or extremely limited presence of such structures in almost all of the control sputa (Fig. S1A). COPD sputum NETs could be clearly identified by comparison with *in vitro* induced NETs (Figs. 4F,G, 5A–D, 6D,E,G,H) and according to the typology provided by the key literature to date [1], [2]. The PMNs contained in these sputa (Fig. 3) represent all steps of the NETotic cascade as defined in the recent literature [2], [13], [15]. These include undisturbed normal sized globular cells with smooth membranes, well defined lobulated nuclei and a clear separation of NE positive cytoplasmic granules and nuclear chromatin, cells at various stages of progressing cytoplasmic and nuclear swelling, vacuolisation and pseudopodial membrane protrusion, chromatin decondensation and histone citrullination, and cells exhibiting membrane rupture and NET release (eg. Figs. 3, 4A). Similarly, the morphology of the NETs contained in COPD sputa is in all relevant aspects, including those of bacterial entrapment (Fig. 4C) and DNase sensitivity (Fig. 2G–I), similar to that of *in vitro* generated NETs as shown in the literature (eg. [36] and by our own present experimentation. High congruence is also evident in comparison to NETs from other chronic inflammatory disorders in the airways and elsewhere. COPD NETs correspond in detail to those found in the sputa of patients with CF [5], [17] and in subgingival plaques from patients with periodontis [37]. This relates also to the features of immunolocalisation of NE, citH3 and PAD4 during the NETotic process and in the generated NETs themselves [2]. The unexpected finding that staining for PAD4 and citH3 at cytoplasmic sites of activated PMNs is just as common as at nuclear sites (Figs. 3C–F) conforms with the principal characteristics of PAD4 localisation in neutrophils and other cell of the innate immune system [38], and with the observation by [39] that H3 deimination during NETosis occurs to a relevant extent in the cytoplasm. The resulting overall picture of COPD sputum NETs is in close agreement with the ‘NET release during pseudopodial crawling’ model of [15]. Examination of COPD NETs collected by bronchoscopy may, as a next step, serve to further determine their direct interaction with airway epithelia and role in loss of respiratory function.

Regarding the general micromorphology of NETs, the present results expand upon the previous (ultra)structural analyses in a number of ways. Perhaps the most important finding in this direction is that TEM of negative stained specimens reveals – to our understanding for the first time in a clear way – that nuclear chromatin decondensation during NETosis is most extensive (Figs. 5, 6) and involves unstacking of nucleosomes down to dimensions much smaller than the 15–17 nm chromatin stretches described by [6], [1], [4]. The present results provide evidence that relevant proportions of the DNA fibres in COPD NETs are comprised of nucleosome-bearing single double-helix molecules in ‘beads-on-a-string’ conformation (Fig. 6G,H), as known from previous experimental TEM analyses of chromosomal DNA [34], [35] and recent atomic force microscopy based models of chromatin structure [40]. ‘Beads-on-a-string’ DNA has as yet been only shown in the dilated perinuclear spaces and cytoplasmic vesicles of human neutrophils during a rapid form of NETosis in response to *in vitro* induction by *Staphylococcus aureus* [32]. The present results, however, suggest that ‘beads-on-a-string’ DNA is routinely released in relevant quantities during ‘normal type’ NETosis, both *in vitro* and *in vivo*, and is thus a common feature of NETs. The additional finding that the nucleosomes on such DNA strings remain all unlabelled after immunogold staining for citH3 (Fig. 6H) indicates that their histone octamers are unaffected by PAD4 mediated arginine deimination. This in turn would mean that the citrullination of histone proteins, a molecular feature regarded as essential for NET formation [9], is in fact a non-quantitative process. This may provide a starting point for exciting new work into the structures and dynamics of NETosis.

Another set of findings from the present work that consolidate and improve the present knowledge on NET structure and its functional implications relates to the abundance of bactericidal proteins and their location within the NETs. The serine protease NE is one of the most powerful molecular weapons that PMNs keep available to degrade engulfed bacteria in the phagolysosome [41]. NE has been shown to contribute to nuclear chromatin decondensation during NETosis [42] and is characteristically found in NETs (eg. [1] including those contained in CF sputum [17], [43]). The present CLSM and immunogold TEM results confirm that NE is indeed also a bulk constituent of NETs derived from COPD sputum. It occurs in high densities in nearly all of the amorphous ‘grid point’ aggregates of these NETs (Fig. 6A), but also at many other, probably non-DNA-associated positions in the sputum matrix (Fig. 2D–F). This pattern is similar to that found for *in vitro* generated NETs (Fig. 6D). However, ambiguity still remains surrounding the extent of the noxious effects of NET-bound NE, especially in the light of recent evidence that experimental NE inhibition does not inhibit the cytotoxicity of NETs [44]. This requires further research.

A similarly ambiguous picture is seen for histones. Being highly cationic, histones have long been attached with an extracellular bactericidal role, specifically in NETs [1], [45]. In this direction, the present results show that citrullinated histone H3 (citH3) positive sites are much less frequent in NETs than NE positive sites (Figs. 6A,B,D,E). This is, to our knowledge, the first experimental evidence of a rather limited occurrence of citH3 in NETs and suggests a similarly limited role in the functions and effects of NETs. This would reaffirm the previous finding that PAD4-mediated citrullination decreases the bactericidal activity of histones, thus confining its role to chromatin decondensation during NET formation instead of improving bacterial killing [8]. However, there is increasing evidence that citrullinated proteins externalised in NETs also act indirectly to reinforce and perpetuate the inflammatory cycle via induction of autoantibodies [46]–[48], thus revaluing the contribution of citH3 to the cumulative effects of histones in NETs. These effects may be further strengthened by the finding that NETs are likely to contain a substantial quantity of non-citrullinated histones on ‘beads-on-a-string’ DNA (Fig. 6G,H). This indicates that the bactericidal capability of NETs, just as their histone-dependent cytotoxicity [44], may be enhanced by a relevant amount of non-citrullinated histones. As these histones are freely exposed on fully dispersed DNA, they are best enabled to establish tight surface contact with both pathogens and epithelial cells (Fig. 6C). This is in agreement with the experimental results of Saffarzadeh and coworkers. [44] showing that histones are main inductors of NET-mediated epithelial and endothelial cell death. This is also in agreement with, and strengthens, the previous finding that the bactericidal effects of NETs are largely eliminated by DNase treatment, and are therefore likely to require microbe entrapment by the extracellular chromatin and exposure to high local concentrations of bactericidal agents [13], [44].

## Conclusions

The present work provides clear morphological evidence that NETs and NETotic PMNs are important constituents of sputa from patients with exacerbated COPD. Comparing the structure of COPD NETs with those of *in vitro* generated NETs, the present work provides a substantial addition to our knowledge of NET micromorphology. It demonstrates that release of nuclear chromatin in ‘beads-on-a-string’ conformation is a common feature of *in vivo* NETosis and exposes potentially highly cytotoxic non-citrullinated core histones to the extracellular environment. This may readily compensate for the low abundance of citrullinated histone H3 (citH3) among the NET-associated proteins as compared to NE, with probable implications for antimicrobial efficiency, host tissue damage and therapeutic targeting.

## Supporting Information

Figure S1 **Controls.** A: Representative example of control sputum devoid of NETs; specimen stained with anti-NE (green) and PI (red). Two intact neutrophils (arrows) and an epithelial cell nucleus (arrowhead) are labelled. B–F: Examples of standard negative controls of antibody reactivity. B–D: Sample from CLSM analysis, DNA stained with PI. B: Primary antibody omitted. Background staining by Dylight 488 flourochrome of secondary antibody is very low. C,D (shown for comparison): Specimens of same sputum sample after regular immunostaining for citH3 (C) and NE (D). The Dylight 488 signal (green) is clearly localised in the target structures. E–G: Immunogold staining for TEM. E: Primary antibody omitted. Background staining by 5 nm gold conjugated to secondary antibody is confined to only very few items (arrow). F,G (shown for comparison): Patterns of gold labelling on specimens of the same sputum sample after regular staining for citH3 (F) and NE (G).
(TIF)

Figure S2 **Isotype controls.** None of the non-specific control antibodies reacts with PMNs or extracellular structures. A: mouse anti-PAD4 IgG2a replaced by BioLegend 400212; B: polyclonal rabbit anti-human NE/citH3 IgG replaced by Sigma I5006; DNA stained with DAPI (A) and PI (B).
(TIF)
