# Supplementary figures and images for: New Aspects on the Structure of Neutrophil Extracellular Traps from Chronic Obstructive Pulmonary Disease and In Vitro Generation
Source: PLoS One. 2014 May 15;9(5):e97784. doi: 10.1371/journal.pone.0097784 (PMC4022649; doi:10.1371/journal.pone.0097784)

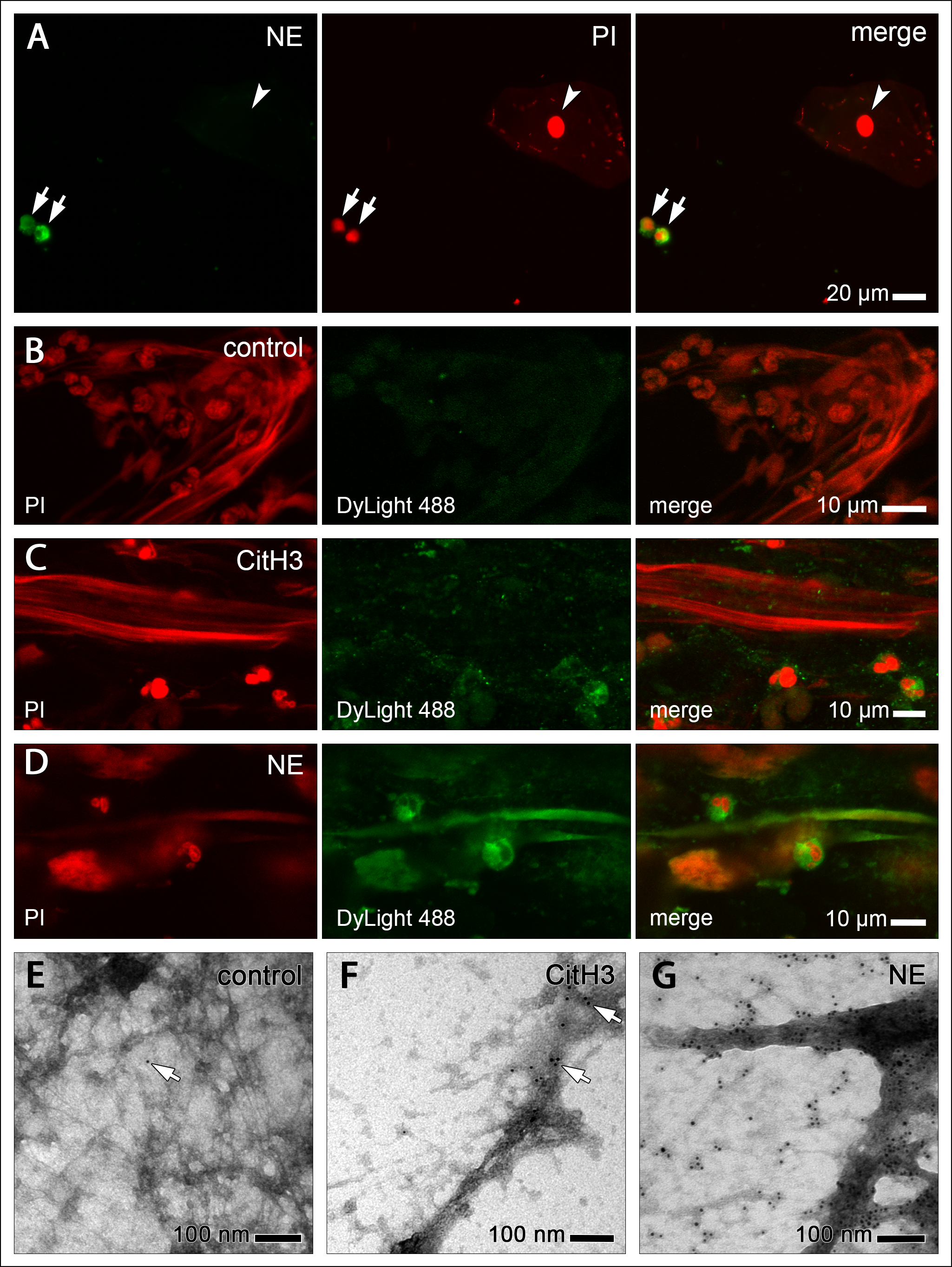

Supplement: Figure S1 — Controls. A: Representative example of control sputum devoid of NETs; specimen stained with anti-NE (green) and PI (red). Two intact neutrophils (arrows) and an epithelial cell nucleus (arrowhead) are labelled. B–F: Examples of standard negative controls of antibody reactivity. B–D: Sample from CLSM analysis, DNA stained with PI. B: Primary antibody omitted. Background staining by Dylight 488 flourochrome of secondary antibody is very low. C,D (shown for comparison): Specimens of same sputum sample after regular immunostaining for citH3 (C) and NE (D). The Dylight 488 signal (green) is clearly localised in the target structures. E–G: Immunogold staining for TEM. E: Primary antibody omitted. Background staining by 5 nm gold conjugated to secondary antibody is confined to only very few items (arrow). F,G (shown for comparison): Patterns of gold labelling on specimens of the same sputum sample after regular staining for citH3 (F) and NE (G). (TIF) [file pone.0097784.s001.tif]

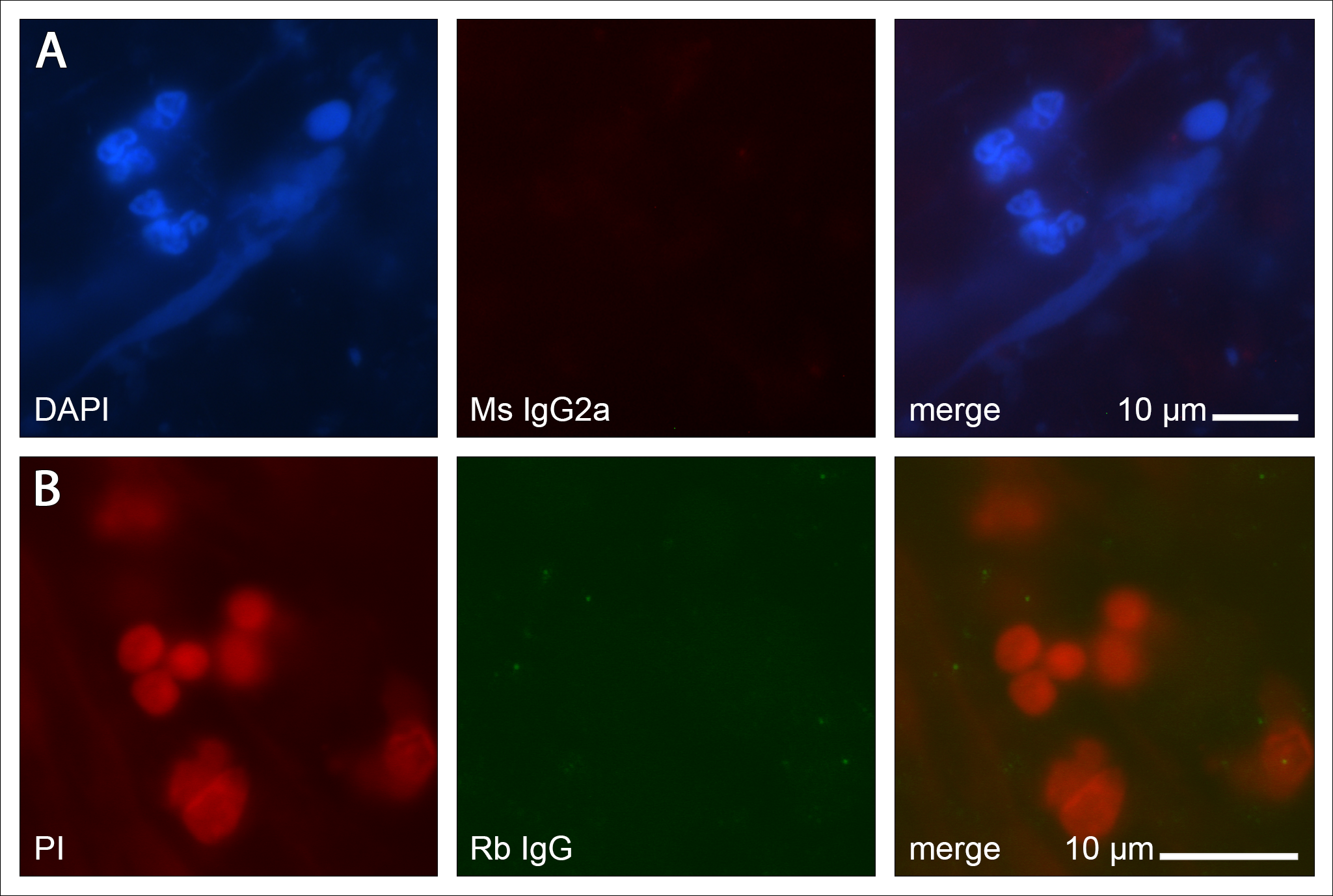

Supplement: Figure S2 — Isotype controls. None of the non-specific control antibodies reacts with PMNs or extracellular structures. A: mouse anti-PAD4 IgG2a replaced by BioLegend 400212; B: polyclonal rabbit anti-human NE/citH3 IgG replaced by Sigma I5006; DNA stained with DAPI (A) and PI (B). (TIF) [file pone.0097784.s002.tif]
